# Value of blood oxygenation level-dependent magnetic resonance imaging in early evaluation of the response and prognosis of esophageal squamous cell carcinoma treated with definitive chemoradiotherapy: a preliminary study

**DOI:** 10.1186/s12880-024-01193-9

**Published:** 2024-01-12

**Authors:** Huanhuan Zheng, Hailong Zhang, Yan Zhu, Xiaolei Wei, Song Liu, Wei Ren

**Affiliations:** 1 Department of Radiology, Nanjing Drum Tower Hospital, The Affiliated Hospital of Nanjing University Medical School, No. 321 Zhongshan Road, Nanjing, 210008 China; 2 The Comprehensive Cancer Center of Drum Tower Hospital, Medical School of Nanjing University and Clinical Cancer Institute of Nanjing University, Nanjing, 210008 China

**Keywords:** Esophageal neoplasms, Blood oxygenation level-dependent, Response, Prognosis, Chemoradiotherapy

## Abstract

**Background:**

To find a useful hypoxia non-invasive biomarker for evaluating early treatment response and prognosis to definitive chemoradiotherapy (dCRT) in patients with esophageal squamous cell carcinoma (ESCC), using blood oxygenation level-dependent (BOLD) magnetic resonance imaging (MRI).

**Methods:**

The R2* values were obtained pre- and 2–3 weeks post-dCRT in 28 patients with ESCC using BOLD MRI. Independent samples t-test (normality) or Mann-Whitney U test (non-normality) was used to compare the differences of R2*-related parameters between the complete response (CR) and the non-CR groups. Diagnostic performance of parameters in predicting response was tested with receiver operating characteristic (ROC) curve analysis. The 3-year overall survival (OS) was evaluated using Kaplan Meier curve, log rank test, and Cox proportional hazards regression analysis.

**Results:**

The post-R2*, ∆R2*, and ∆%R2* in the CR group were significantly higher than those in the non-CR group (*P* = 0.002, 0.003, and 0.006, respectively). The R2*-related parameters showed good prediction of tumor response, with AUC ranging from 0.813 to 0.829. The 3-year OS rate in patients with ∆R2* >-7.54 s^− 1^ or CR were significantly longer than those with ∆R2* ≤ -7.54 s^− 1^ (72.37% vs. 0.00%; Hazard ratio, HR = 0.196; 95% confidence interval, 95% CI = 0.047–0.807; *P* = 0.024) or non-CR (76.47% vs. 29.27%; HR = 0.238, 95% CI = 0.059–0.963; *P* = 0.044).

**Conclusions:**

The preliminary results demonstrated that the R2* value might be a useful hypoxia non-invasive biomarker for assessing response and prognosis of ESCC treated with dCRT. BOLD MRI might be used as a potential tool for evaluating tumor oxygenation metabolism, which is routinely applied in clinical practice and beneficial to clinical decision-making. A large sample size was needed for further follow-up studies to confirm the findings.

**Supplementary Information:**

The online version contains supplementary material available at 10.1186/s12880-024-01193-9.

## Background

Esophageal cancer is one of the leading causes of cancer-related deaths worldwide, with relatively low survival rate [1], and esophageal squamous cell carcinoma (ESCC) is the main pathological type in China [2]. Definitive chemoradiotherapy (dCRT) is the standard of care for inoperable patients with ESCC, which has a comparable survival and quality of life to surgical resection for favorable responders [3, 4]. However, the therapeutic response is affected by tumor oxygenation. Since hypoxia plays an important role in tumor angiogenesis and metastasis, it can increase resistance to chemoradiotherapy [5]. It has been confirmed that the hypoxic part of tumor is insensitive to chemoradiotherapy, which is correlated with poor prognosis [6]. Therefore, early monitoring of the status of tumor oxygenation is crucial to evaluate the treatment response and prognosis of ESCC treated with dCRT.

The traditional imaging methods, such as barium esophagography and computed tomography (CT), for evaluating the therapeutic response of ESCC are based on the tumor morphological changes after treatment, and do not evaluate tumor oxygenation. Although positron emission tomography (PET) can be used for hypoxia imaging to evaluate the treatment response to chemoradiotherapy in patients [7], high cost and radiation exposure limit its widespread application.

Blood oxygenation level-dependent (BOLD) magnetic resonance imaging (MRI) has been used as a noninvasive tool for assessing the tissue oxygen metabolism [8, 9]. Since deoxyhemoglobin shortens T2* relaxation time, R2* (= 1/T2* relaxation time) can reflect the relative content of deoxyhemoglobin in tissue and is regarded as a quantitative parameter of signal attenuation in BOLD images. Currently, R2* value has been widely utilized in assessing cerebrovascular reactivity [10], muscle activation [11, 12], renal function [13, 14], blood perfusion and histopathological status of tumors [15–18]. Especially, BOLD MRI has been demonstrated to be effective in evaluating the treatment response and prognosis to chemoradiotherapy in various tumors, such as prostate [19], cervical [20, 21] and breast cancer [22]. A preliminary study has reported that blood oxygenation T2* value is a useful quantitative indicator for differentiating preoperative stage of ESCC [20]. However, to the best of our knowledge, BOLD MRI has not been used for assessing the therapeutic response to dCRT and prognosis in ESCC. Tumor hypoxia is an independent prognostic factor, which is significantly associated with increased resistance to treatment and decreased cancer-free survival [6]. Given that BOLD MRI can assess the tissue oxygenation status, we hypothesized that the R2* value may be a hypoxia imaging predictor for response and prognosis of ESCC treated with dCRT.

Therefore, this study aimed to investigate the value of BOLD MRI in early evaluation of the treatment response and prognosis for dCRT in ESCC patients.

## Methods

### Patients

**Fig. 1:**
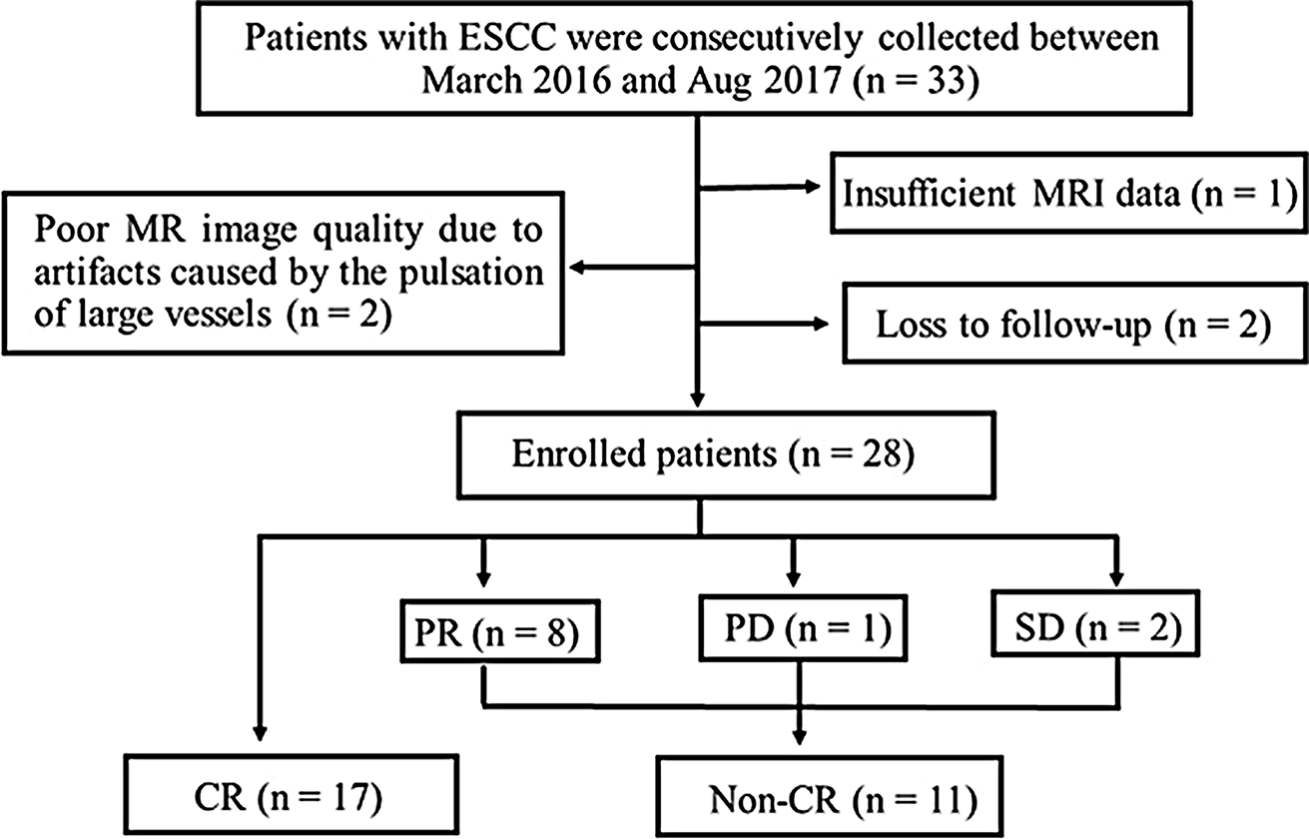
The flowchart of the patients enrolled in this study. CR, complete response; PR, partial response; PD, progressive disease; SD, stable disease

**Fig. 2:**
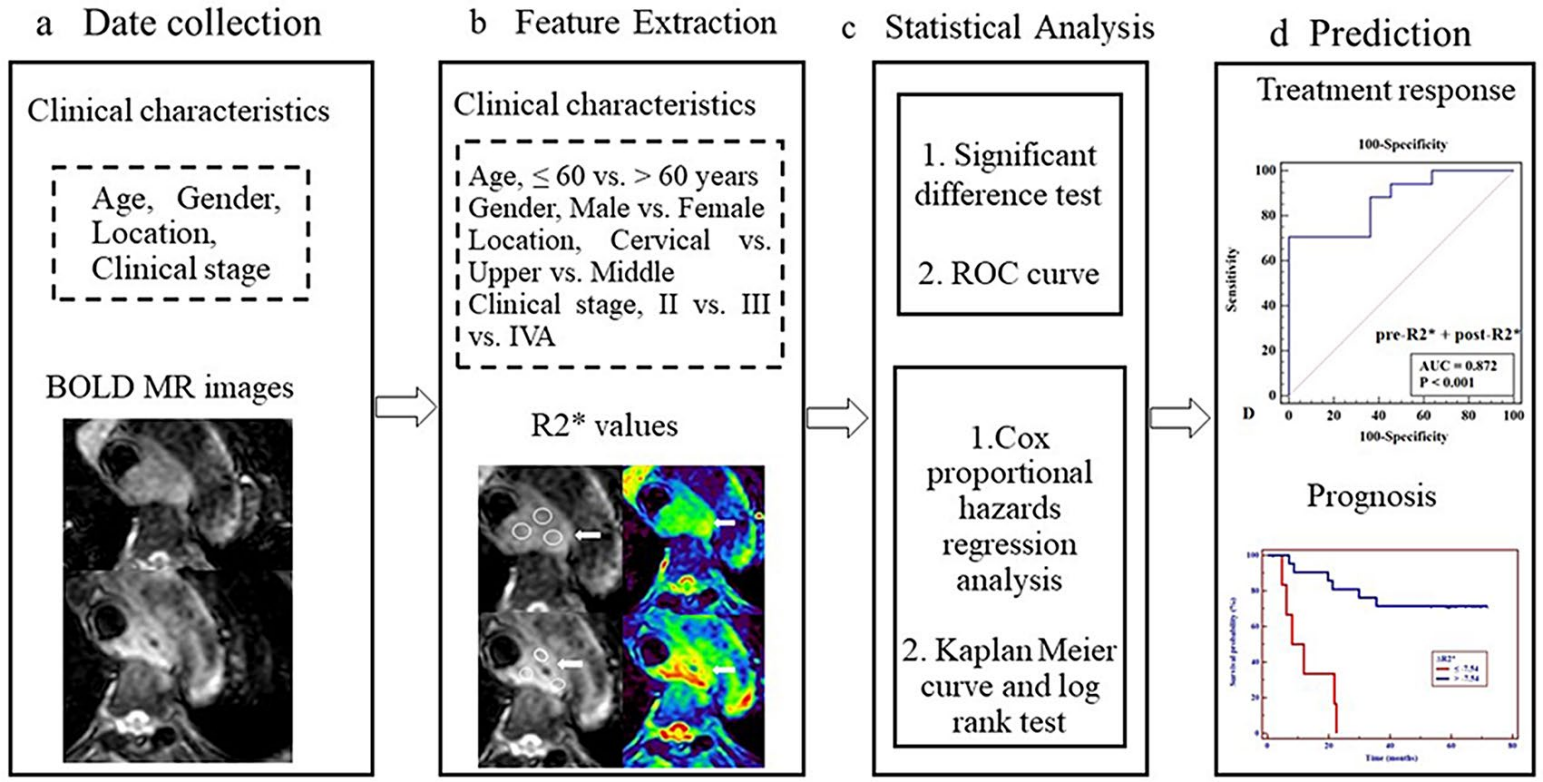
The flowchart of study design. (**a**) Clinical characteristics and BOLD images of patients with ESCC who were treated with definitive chemoradiotherapy were collected. (**b**) Clinical characteristics and R2* value-related parameters were extracted. (**c**) Statistical analysis. (**d**) Diagnostic performance for predicting response and prognosis were obtained by ROC curve analysis, Cox proportional hazards regression analysis, Kaplan Meier curve and log rank test, respectively. BOLD, blood oxygenation level-dependent; MR, magnetic resonance; ROC, receiver operating characteristic

This retrospective study was approved by our institutional review board (IRB, 2022-521-03) and followed the ethical standards of the World Medical Association (Declaration of Helsinki). The informed consent was waived for patients. The data of patients with ESCC who underwent BOLD MRI examination and completed dCRT between March 2016 and Aug 2017 were collected. The inclusion criteria were as follows: (1) diagnosis of esophageal cancer confirmed by endoscopic biopsy; (2) no local or systematic treatment before MRI scan. The exclusion criteria were as follows: (1) insufficient MRI data (*n* = 1); (2) poor MR image quality due to artifacts caused by the pulsation of large vessels (*n* = 2); and (3) Lost to follow-up (*n* = 2). A total of 33 consecutive patients were initially enrolled. Finally, 28 patients (23 men and five women; mean age, 64.25 ± 7.65 years; range, 45–80 years) formed the study cohort (Fig. 1). The flowchart of study design is shown in Fig. 2.

### MRI examination

All patients underwent MRI examinations pre-dCRT (within five days before dCRT) and post-dCRT (2–3 weeks after the start of dCRT) on a 3.0T MR scanner (Ingenia 3.0 T; Philips Medical Systems, Best, the Netherlands), with a 32-channel dStream Torso coil. Patients were trained in breath-holding before MRI examination and instructed to avoid swallowing during the scan.

Axial BOLD MR images were obtained using the respiratory-triggered multiple fast field echo (mFFE) sequence. The scan parameters were as follows: repetition time (TR), 100 ms; range of echo time (TE), 4–40 ms; flip angle, 27°; slice thickness, 5 mm; interslice gap, 1.5 mm. field of view (FOV), 400 × 400 mm; slices, 12. The corresponding T2* mappings were automatically generated. Axial T2-weighted (T2W) images were collected as high-resolution structural maps using a respiratory-triggered turbo spin-echo sequence (TR, 1000 msec; TE, 80 msec; matrix, 260 × 228; section thickness, 5 mm; gap, 0.5 mm; field of view (FOV), 390 × 390 mm; slices, 32).

### Image analysis

All MR images were transmitted into the workstation (Extended MR WorkSpace 2.6.3.5; Philips Medical Systems) and were reviewed together by two radiologists (X.X., X.X.) with eight and 11 years of experience in MRI, respectively, who were blinded to clinicopathological information of the patients.

The location of esophageal cancer was identified on T2-weighted (T2W) images. Esophageal cancer usually presents as a thickening of the esophageal wall or a mass lesion. On T2W images, normal esophageal mucosa appeared isointense, submucosa showed hyperintense and muscularis propria showed iso to hypointense [23]. The tumor appeared slightly hypointense compared with mucosa and muscularis propria, but hyperintense compared with submucosa.

**Fig. 3:**
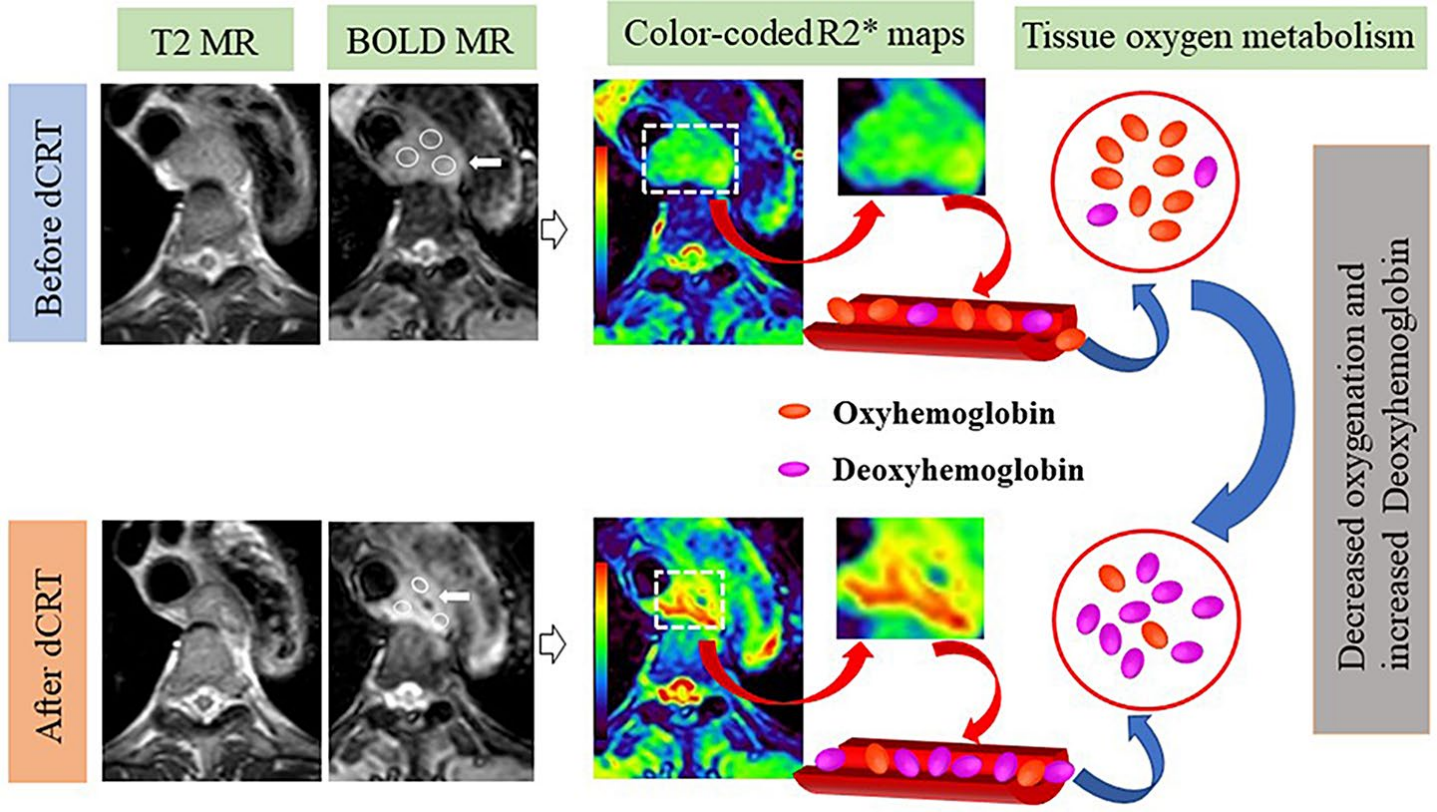
A 62-year-old man with ESCC received definitive chemoradiotherapy (dCRT) and achieved CR. Before dCRT, axial T2W and BOLD images showed a mass lesion at the upper thoracic esophagus (white arrow). On the corresponding color-coded R2* maps, blue-green represents low-medium R2* values, reflecting low-medium concentration of deoxyhemoglobin. Three oval regions of interest of the lesion were drawn, with a mean value of 30.58 s^-1^. Approximately 2–3 weeks after dCRT, T2W, BOLD, and corresponding color-coded R2* maps showed that the mass had shrunken (white arrow). Red represents high R2* values, reflecting high concentration of deoxyhemoglobin. The R2* value of the lesion increased to 56.79 s^-1^

The BOLD MR images were loaded into SPIN software (Magnetic Resonance Innovations Inc., Detroit, Michigan), and the corresponding color-coded R2* maps and T2* mapping of the lesion was automatically generated. After referring to corresponding T2W images, three oval region of interests (ROIs) were drawn by the two radiologists at the largest tumor area slice on BOLD MR images and were simultaneously copied to T2* mapping images. The three ROIs covered the solid part of the tumor as much as possible to avoid macroscopically visible necrosis or cystic degeneration and adjacent tissues (Fig. 3). If the lesion had majorly shrunken after dCRT, the ROIs were placed at the same region of pre-treated tumor after referring to the previous MR images [24]. The average value of three ROIs were calculated as the final data. Based on the ROI, the T2* value of the lesion was acquired. Furthermore, the R2* value of the lesion was calculated as follows: R2* = 1/T2*. The changes of R2* values (∆R2* and ∆%R2*) were also calculated. For instance, ∆R2* = post-R2*- pre-R2*, and ∆%R2* = ∆R2* / pre-R2* × 100%, where pre-R2* and post-R2* were R2* values before and 2–3 weeks after dCRT, respectively.

### Treatment options

All patients were treated with dCRT. The prescription dose of intensity-modulated radiation therapy (IMRT) was 60–66 Gy in 30–33 fractions (2 Gy for each fraction). The prescribed concurrent chemotherapy was performed with weekly regimen of paclitaxel liposome (50 mg/m^2^) and nedaplatin (25 mg/m^2^) for 4–6 weeks.

### Response evaluation and follow-up

Objective response to treatment was assessed one month after the end of dCRT according to the Response Evaluation Criteria in Solid Tumors (RECIST), including complete response (CR), partial response (PR), progressive disease (PD) and stable disease (SD). All patients were followed-up at 1, 3, and 6 months, and then every 6 months after treatment until death. The time from the date of dCRT initiation to death due to any cause was recorded as overall survival (OS). The follow-up was completed on March 25, 2022.

### Statistical analysis

The Shapiro-Wilk test was used to check the normality assumption for all parameters. According to the results of Shapiro-Wilk test (Supplemental Table 1), the variations of R2^*^ at pre- and post-dCRT were observed by Wilcoxon test. Independent samples t-test (normality) or Mann-Whitney U test (non-normality) was used to compare differences of parameters between the CR and non-CR groups. Clinical characteristics of different groups were compared using Fisher’s exact tests. The diagnostic performance of the parameters in predicting treatment response was tested with receiver operating characteristic (ROC) curve analysis. The 3-year OS of variables were compared by Kaplan Meier curve and log rank test. Variables with *P* < 0.05 in the univariate analysis were finally included in the multivariable analysis and a stepwise backward method was used in Cox proportional hazards regression analysis. Statistical analyses were performed with SPSS (v.22.0 for Microsoft Windows x64, SPSS, Chicago, IL). ROC analysis was performed using MedCalc Statistical Software version 19 (MedCalc Software bvba, Ostend, Belgium; https://www.medcalc.org; 2019). A two-tailed P value < 0.05 was considered as statistically significant.

## Results

### Patient characteristics

Among 28 patients with ESCC, the cases with CR, PR, PD and SD were 17 (60.71%), eight (28.57%), one (3.57%) and two (7.14%), respectively. PR, PD and SD patients were classified as the non-CR group. The median follow-up time was 61.1 (2–71) months. Moreover, 13 (46.43%) patients died of ESCC, one (3.57%) died of respiratory failure caused by radioactive pneumonia and 14 (50.00%) patients survived. The baseline characteristics of all patients are listed in Table 1. There was no significant difference in the baseline characteristics between the CR and non-CR groups (all *P* > 0.05).

**Table 1:** Clinical characteristics of patients

Characteristics	All Patients	non-CR	CR	*P*
	(*n* = 28)	(*n* = 11)	(*n* = 17)	
**Age at diagnosis (years)**				1.000
≤ 60	9 (32.14%)	3 (10.71%)	6 (21.43%)	
> 60	19 (67.86%)	8 (28.57%)	11 (39.29%)	
**Gender**				1.000
Male	5 (17.86%)	2 (7.14%)	3 (10.71%)	
Female	23 (82.14%)	9 (32.14%)	14 (50.00%)	
**Location**				0.062
Cervical	7 (25.00%)	1 (3.57%)	6 (21.43%)	
Upper	11 (39.29%)	3 (10.71%)	8 (28.57%)	
Middle	10 (35.71%)	7 (25.00%)	3 (10.71%)	
**Clinical stage**				0.424
II	10 (35.71%)	5 (17.86%)	5 (17.86%)	
III	12 (42.86%)	3 (10.71%)	9 (32.14%)	
IVA	6 (21.43%)	3 (10.71%)	3 (10.71%)	

*Note*: Data are presented as median (range) or n (%). Location and clinical stage before treatment were according to the American Joint Committee on Cancer (8th edition). CR = complete response

### Comparison of R2* values at pre-dCRT and post-dCRT

**Fig. 4:**
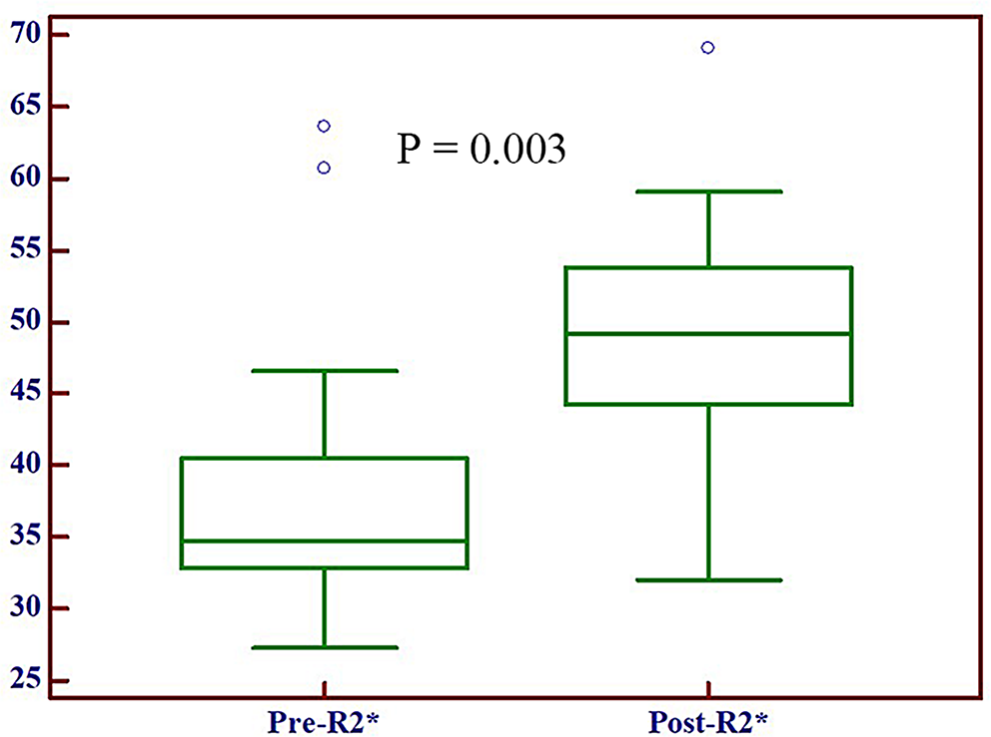
Box-whisker plots of R2* values in ESCC at pre- and post-dCRT in the CR group. The horizontal line through each box represents the median value and the box represents data of 95% confidence intervals. Graph shows that R2* values of esophageal cancer had significantly increased at 2–3 weeks post-dCRT in the CR group (*P* = 0.003)

In the CR group, the R2* values were significantly increased from 38.20 ± 10.26 s^− 1^ to 48.89 ± 8.19 s^− 1^ (*P* = 0.003) after 2–3 weeks of dCRT (Fig. 4). In contrast, the R2* values were decreased by 37.66 ± 8.12 s^− 1^ from 40.61 ± 10.18 s^− 1^ in the non-CR group (*P* = 0.286).

### Differences of R2* values between the CR and non-CR groups

Table 2 shows the differences of R2* values between the CR and non-CR groups before and after dCRT. The post-R2*, ∆R2* and ∆%R2* values in the CR group were significantly higher than those in the non-CR group (*P* = 0.002, 0.003, and 0.006, respectively). While there was no significant difference in pre-R2* between the CR and non-CR groups (*P* = 0.264).

**Table 2:** Differences in the R2* values between the CR and the non-CR groups

Parameters	Non-CR	CR	*P*
Pre-R2* (s^− 1^)	38.61 (13.52)	34.75 (8.98)	0.264
Post-R2* (s^− 1^)	37.66 ± 8.12	48.99 ± 8.82	0.002
∆R2*(s^− 1^)	-2.95 ± 9.59	10.78 ± 11.44	0.003
∆%R2*	-2.85 ± 29.21	33.87 ± 33.00	0.006

*Note*: Data are presented as mean ± standard deviation (normality) or median (interquartile range) (non-normality). CR = complete response. P values with independent samples t-test (normality) or Mann-Whitney U test (non-normality)

### Prediction of early tumor response

**Fig. 5:**
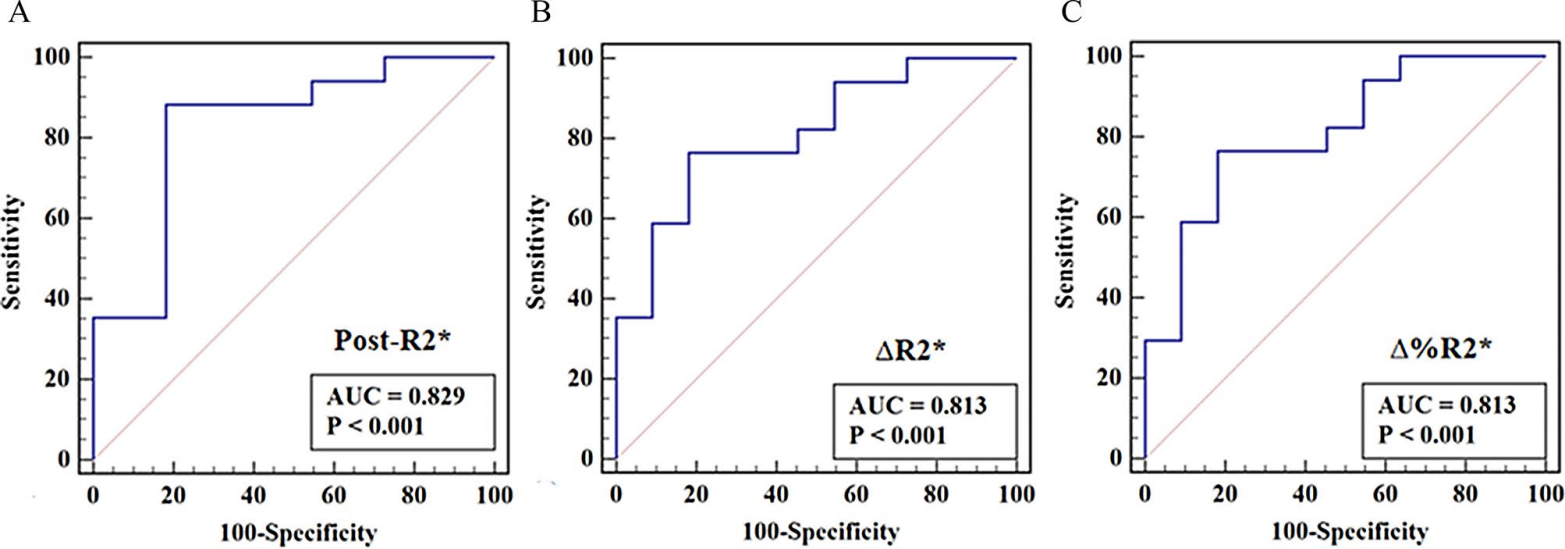
ROC curves of R2* values in early identification of CR from non-CR in ESCC. The post-R2* (2–3 weeks post-dCRT), ∆R2*, ∆%R2* and the fitting parameter (pre-R2* combined with post-R2*) values showed good prediction performance, yielding an AUC of 0.829, 0.813, and 0.813 (**A**∆**C**), respectively

Table 3 shows the diagnostic performance of R2* values in differentiating CR from non-CR patients. After 2–3 weeks of dCRT, the post-R2*, ∆R2, and ∆%R2* showed good prediction of tumor response with an area under the curve (AUC) of 0.829, 0.813, and 0.813, respectively (Fig. 5).

**Table 3:** Diagnostic performance of R2* values in differentiating CR from non-CR in patients with ESCC

Parameters	Cutoff	Sensitivity	Specificity	Accuracy	AUC
Pre-R2* (s^− 1^)	≤ 36.60	64.71%	72.73%	67.86%	0.631
Post-R2* (s^− 1^)	> 39.71	88.24%	81.82%	85.72%	0.829
∆R2*(s^− 1^)	> 2.76	76.47%	81.82%	78.57%	0.813
∆%R2*	> 7.85	76.47%	81.82%	78.57%	0.813

*Note*: CR = complete response; AUC = area under the curve

### Survival analysis

**Fig. 6:**
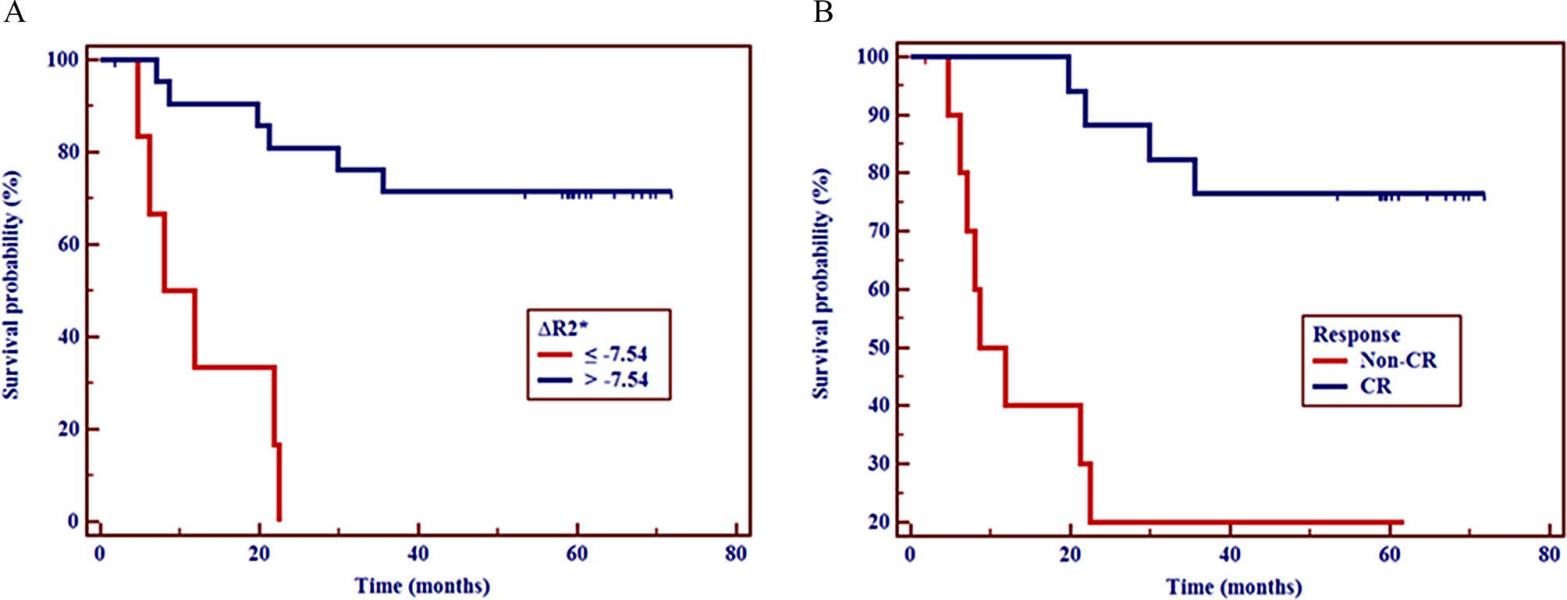
Kaplan–Meier survival curves of overall survival (OS) in patients. Kaplan–Meier survival curves showing 3-year OS for patients with a ∆R2* value ≤ -7.54 s^-1^ compared to those with a ∆R2* value > -7.54 s^-1^ (**A**), and patients with a CR compared to those with a non-CR (**B**). Both P values obtained by log rank test were < 0.001

The overall 1-year, 2-year, and 3-year survival rates after dCRT were 75.00%, 60.71%, and 53.57%, respectively. We used X-tile to obtain the optimal thresholds of the R2*-related parameters [25]. According to the optimal thresholds, R2*-related parameters were divided into two groups (Table 4). The 3-year OS rate of patients with ∆R2* >-7.54 s^− 1^ were significantly longer than those with ∆R2* ≤ -7.54 s^− 1^ (72.37% vs. 0.00%; hazard ratio, HR = 0.196; 95% confidence interval, 95% CI = 0.047–0.807; *P* = 0.024), based on multivariate analysis (Fig. 6). The ESCC patients with a CR were associated with better survival prognosis of dCRT (HR = 0.238, 95% CI = 0.059–0.963; *P* = 0.044).

**Table 4:** Univariate and multivariate analyses for 3-year overall survival

Variables	Univariate analysis			Multivariate analysis		
	HR	95% CI	P	HR	95% CI	*P*
Age (> 60 year)	1.452	0.436–4.832	0.543			
Gender (man)	0.287	0.072–1.142	0.091			
^§^Location	0.971	0.290–3.250	0.962			
^§^Clinical stage	1.270	0.390–4.132	0.691			
Pre-R2*(> 42.18 s^− 1^)	9.175	2.284–36.862	0.002			
Post-R2* (> 35.16 s^− 1^)	0.092	0.018–0.483	0.005			
∆R2* (>-7.54 s^− 1^)	0.018	0.003–0.112	< 0.001	0.196	0.047–0.807	0.024
∆%R2* (>-12.77)	0.018	0.003–0.112	< 0.001			
Response (CR)	0.078	0.020–0.308	< 0.0011	0.238	0.059–0.963	0.044

*Note*: §, Location, Cervical + Upper vs. Middle; Clinical stage, II vs. III + IVA; HR, Hazard ratio; 95% CI, 95% confidence interval

## Discussion

Hypoxia plays a vital role in tumor microenvironment by allowing development and maintenance of cancer cells [26]. Hypoxia is a major hindrance for effective anti-cancer therapy and the main reason for failure of most anti-cancer drugs and radiotherapy. Therefore, early monitoring of hypoxic status in tumors can predict the efficacy during treatment, which can help with timely adjustment of treatment plans to avoid extra chemoradiotherapy toxicity and economic burden. BOLD MRI can non-invasively evaluate tissue oxygenation metabolism, and R2* quantification reflects the changes of deoxyhemoglobin content. This study found that the post-R2* and change of R2* values in the CR group were significantly higher than those in the non-CR group, and ∆R2* was an independent prognostic factor of ESCC after dCRT. These findings suggested that BOLD MRI might be useful in evaluating early tumor response and forecasting the prognosis of patients with ESCC who underwent dCRT, and monitoring the changes of tumor oxygenation during treatment.

We found that the R2* values significantly increased after dCRT in CR patients, which might be explained by the increase of local deoxyhemoglobin content due to the reduction of neovascularization in the tumor after treatment, as well as the decrease of vascular permeability and blood flow [20]. A study on dynamic contrast-enhanced MRI also revealed that the K^trans^ and K^ep^ values, which reflected the tumor microcirculation perfusion and vessel permeability, decreased significantly after chemoradiotherapy in patients with ESCC [27]. Increase in R2* value was also found after chemoradiotherapy in cervical cancer [20] and breast cancer [22]. However, in this study, there was no significant difference in the R2* value of non-CR patients before and after treatment. The results suggested that the tumor was hypoxic after treatment, and patients with increased R2* values might be more sensitive to dCRT.

Previous studies had reported that R2* values might have the potential as an imaging biomarker for predicting breast cancer response to treatment [28]. This study showed that the R2* values were significantly higher in the CR group than in the non-CR group after 2–3 weeks of dCRT. Patients in the CR group were more likely to exhibit tumor shrinkage and fibrosis after dCRT, which may lead to local tumor hypoxia with higher R2* value. This means that early change in R2* values might indicate short-term outcomes for ESCC. Therefore, the post-R2* differences in this study were clinically relevant. The findings showed that higher R2*-related parameter values in the early stage after dCRT showed good response. Once post-R2* and change in R2* values were known, it might be able to screen out patients with CR as early as possible after dCRT (2-3weeks after treatment), which might help to provide reference value for selecting appropriate consolidation treatment for patients achieving non-CR.

Oxygenation of tumors before treatment is important for disease control [28], and pretreatment R2* value was a significant independent predictor of progression and survival in patients [21]. In this study, patients with high pre-R2* had significantly worse survival than those with low pre-R2* (3-year OS rate, 0.00% vs. 76.19%; *P* = 0.002) in the univariate analysis, but it was not an independent predictor in the multivariate analysis. Moreover, a high change of R2* value (∆R2*) was an independent prognostic factor of ESCC after dCRT. However, the small sample size and lack of confounder (age, gender, location, and clinical staging) control made the final statistical significance of our results challenging. Therefore, ∆R2* might be unstable in evaluating prognosis. In addition, early treatment response had an impact on the prognosis of esophageal cancer patients. It might be explained by the fact that non-CR patients with residual tumor were more susceptible to recurrence and metastasis, resulting in a lower survival rate. ∆R2* and treatment response might be helpful in assessing prognosis, but needed to be validated with a large sample size.

In interpreting our findings, several limitations must be taken into account. This study had several limitations. First, the main limitation of this study was the relatively small sample size, which might not be able to strongly support definitive statements about the diagnostic efficacy of our findings. In clinical practice, BOLD MRI was not mandatory for the patients with ESCC, and our inclusion criteria were strict, which resulting in a small sample size of eligible cases. The primary aim of this study was to preliminarily attempt to find an effective non-invasive evaluation biomarker for dCRT, hoping to provide a feasibility foundation for further research by expanding the sample size. Second, there was a lack of control for important confounders in survival analysis. Due to the small sample size in this study, it was not possible to include too many parameters in the multivariate analysis. Nevertheless, in univariate analysis, the important confounders (age, gender, location, and clinical stage) were balanced among the different groups without significant statistical differences. If controlling for multiple hypothesis tests, some of our findings might not meet statistically significant. Hence, the findings might have limited utility for prognosis. Lastly, besides oxygenation, R2* value is also affected by blood volume, blood flow, hemoglobin, etc. Therefore, future studies will be conducted by expanding the sample size, controlling for important confounders, and exploring the mechanism of R2* value reflecting hypoxia to confirm the findings.

## Conclusion

This preliminary study demonstrated that the R2* value might be a useful hypoxia non-invasive biomarkerfor assessing response and prognosis of ESCC treated with dCRT. BOLD MRI might be used as a potential tool for evaluating tumor oxygenation metabolism, which is routinely applied in clinical practice and beneficial to clinical decision-making. A large sample size was needed for further follow-up studies to confirm the findings.

### Electronic supplementary material

Below is the link to the electronic supplementary material.

**Supplementary Material 1: Supplemental Table 1.** Comparison of P values of R2*-related parameters between non-CR and CR patients with Shapiro-Wilk test for normality assumption

## Data Availability

Data and material in the study are available from the corresponding author on reasonable request.

## Abbreviations

ESCC: Esophageal squamous cell carcinomas
dCRT: Definitive chemoradiotherapy
MRI: Magnetic resonance imaging
BOLD: Blood oxygenation level-dependent
T2W: T2-weighted
TR: Repetition time
TE: Echo time
FOV: Field of view
mFFE: Multiple fast field echo
ROIs: Region of interests
IMRT: Intensity-modulated radiation therapy
RECIST: Response Evaluation Criteria in Solid Tumors
CR: Complete response
PR: Partial response
PD: Progressive disease
SD: Stable disease
OS: Overall survival
ROC: Receiver operating characteristic
AUC: Area under the curve
HR: Hazard ratio
95%CI: 95% confidence interval
