# Supplementary material for: Value of blood oxygenation level-dependent magnetic resonance imaging in early evaluation of the response and prognosis of esophageal squamous cell carcinoma treated with definitive chemoradiotherapy: a preliminary study
Source: BMC Med Imaging. 2024 Jan 12;24:18. doi: 10.1186/s12880-024-01193-9 (PMC10787410; doi:10.1186/s12880-024-01193-9)
Supplement: Supplementary file 1 — Supplementary Material 1: Supplemental Table 1. Comparison of P values of R2*-related parameters between non-CR and CR patients with Shapiro-Wilk test for normality assumption [file 12880_2024_1193_MOESM1_ESM.docx]

**Supplemental Table 1.** Comparison of P values of R2*-related parameters between non-CR and CR patients with Shapiro-Wilk test for normality assumption

| Parameters | non-CR | CR |
| --- | --- | --- |
| Pre-R2* | 0.922 | 0.003 |
| Post-R2* | 0.195 | 0.991 |
| ∆R2* | 0.664 | 0.980 |
| ∆%R2* | 0.128 | 0.502 |

Note: CR, complete response; P < 0.05, which denies the normality assumption of the parameter.
